# Complete mitochondrial genomes of two Evacanthini species (Hemiptera, Cicadellidae, Evacanthinae) with phylogenetic implications

**DOI:** 10.3897/zookeys.1292.197902

**Published:** 2026-09-17

**Authors:** Yihan Li, Lina Jiang, Sai Jiang, Yihong Guo, Ran Li, Jichun Xing, Bin Zhang, Yujian Li

**Affiliations:** 1 School of Life Sciences, Qufu Normal University, Qufu, 273165, Shandong Province, China School of Life Sciences, Qufu Normal University Qufu China; 2 Shandong Key Laboratory of Wetland Ecology and Biodiversity Conservation in the Lower Yellow River, Qufu, 273165, Shandong Province, China Shandong Key Laboratory of Wetland Ecology and Biodiversity Conservation in the Lower Yellow River Qufu China; 3 Institute of Entomology, Guizhou University, Guiyang, 550025, Guizhou Province, China Institute of Entomology, Guizhou University Guiyang China; 4 College of Life Sciences & Technology, Inner Mongolia Normal University, Hohhot, 010022, Inner Mongolia Autonomous Region, China College of Life Sciences & Technology, Inner Mongolia Normal University Hohhot China; 5 Key Laboratory of Biodiversity conservation and Sustainable utilization for College and University of Inner Mongolia Autonomous Region, Hohhot, 010022, Inner Mongolia Autonomous Region, China Key Laboratory of Biodiversity conservation and Sustainable utilization for College and University of Inner Mongolia Autonomous Region Hohhot China

**Keywords:** Codon usage bias, leafhoppers, mitogenome, monophyly, non-monophyly, PCGs, phylogeny, rRNAs

## Abstract

The leafhopper tribe Evacanthini, a core lineage within the morphologically diverse subfamily Evacanthinae, provides key insight into the classification, evolutionary history, and molecular evolution of this group. We sequenced and annotated the complete mitochondrial genomes of *Taperus fasciatus* Li & Wang, 1994 and *Onukia* sp. and reconstructed phylogenetic relationships using a concatenated dataset comprising 13 protein-coding genes (PCGs) and two ribosomal RNA genes (rRNAs) with maximum likelihood (ML) and Bayesian inference (BI) methods. The dataset included 38 Evacanthini species and two outgroups from Nirvanini, clarifying the phylogenetic positions of each species and enriching genomic resources for the tribe. Meanwhile, to further test the genus *Onukia* Matsumura, 1912 for non-monophyly as suggested by Wang et al. (2017), we constructed an additional ML tree based on partial *cox1* sequences from 36 species. The results confirmed that *Onukia* is non-monophyletic, with its species scattered across multiple branches rather than forming a single cohesive clade. These findings support the monophyly of Evacanthini while revealing complex evolutionary relationships within *Onukia*, providing robust molecular evidence for future studies on systematics, taxonomy, and evolution of this agriculturally significant group.

## Introduction

The leafhopper subfamily Evacanthinae (Hemiptera: Cicadellidae) represents an early-diverging and morphologically diverse lineage (Dietrich et al. 2017; Hu et al. 2022), traditionally comprising four recognized tribes: Nirvanini, Balbillini, Evacanthini, and Pagaroniini (Dietrich 2004). Based on integrated morphological and molecular phylogenetic evidence, Wang et al. (2017) proposed Pentoffiini as the fifth tribe within Evacanthinae, expanding the tribal classification of this subfamily to five recognized tribes. Among these, the tribe Evacanthini constitutes a core lineage, encompassing multiple genera that exhibit pronounced variation in external morphology, wing venation, and male genital structures (Dietrich 2004; Wang et al. 2017). Although these morphological traits provide valuable taxonomic characters, they have historically complicated efforts to resolve evolutionary relationships within the tribe and its position relative to other Evacanthinae lineages. Consequently, integrative phylogenetic analyses that combine molecular and morphological data are essential to clarify the evolutionary history, intergeneric relationships, and divergence patterns of Evacanthini.

Molecular phylogenetic approaches, particularly those based on mitochondrial genomes, have proven highly effective in resolving complex relationships in leafhoppers. Complete mitogenomes offer several advantages, including high mutation rates, maternal inheritance, and conserved gene content, which facilitate the reconstruction of both deep and shallow phylogenetic splits (Wilson et al. 1985; Wolstenholme 1992; Taanman 1999; Ballard and Whitlock 2004; Cameron 2014; Du et al. 2021; Jiang et al. 2022). In addition, analyses of codon usage, tRNA secondary structures, and non-coding regions can provide supplementary phylogenetic signals, improving the resolution of closely related taxa. Previous studies have largely supported the monophyly of Evacanthini (Wang et al. 2017; Jiang et al. 2024; Wang et al. 2024); however, the genus *Onukia* remains taxonomically problematic. Morphological observations and limited *cox1* sequence data suggest that *Onukia* may not form a single cohesive lineage, indicating potential non-monophyly within the tribe (Wang et al. 2017). Resolving this issue is crucial for accurate genus-level classification and for understanding evolutionary patterns within the subfamily.

In this context, the objectives of the present study are threefold: (1) to characterize the mitochondrial genome structures, nucleotide composition, codon usage patterns, tRNA secondary structures, and non-coding regions of two representative species, and to compare these features with closely related taxa (Du et al. 2021; Wang et al. 2021); (2) to assess the monophyly of Evacanthini and examine intergeneric relationships, thereby clarifying the phylogenetic positions of *Taperus fasciatus* and *Onukia* sp. within the subfamily; and (3) to test the non-monophyly hypothesis of *Onukia* (Wang et al. 2017). By integrating comprehensive mitogenomic and phylogenetic analyses, this study provides a robust framework for understanding the evolutionary history of Evacanthini, helps resolve genus-level relationships, and offers molecular evidence to inform future systematics and taxonomy of this agriculturally significant group (Aguin-Pombo et al. 2007; Chen et al. 2012).

## Materials and methods

### Sample collection and identification

Adult specimens of *T. fasciatus* and *Onukia* sp. were collected from Guilin City, Guangxi Zhuang Autonomous Region, China (26°06'N, 109°48'E) (Table 1). Specimens were collected from natural habitats in the wild, immediately preserved in 100% ethanol, and stored long-term at -80 °C for DNA extraction. Species identification was primarily based on morphological descriptions in the *Fauna Sinica* and relevant authoritative taxonomic literature (Li et al. 2020). To minimize potential interference from gut microbial DNA in subsequent mitochondrial genome sequencing and assembly, the abdomens of all specimens were removed prior to DNA extraction. The morphological voucher specimens, including the remaining body parts and dissected male genitalia, were preserved in the School of Life Sciences, Qufu Normal University, Shandong Province, China.

**Table 1.** Sequences used in this study.

**Genus**	**Organism**	**GenBank accession numbers**	**Length**
* Apphia *	*Apphia albida*	PX412734.1	15,327
	*Apphia albonotata*	PV802420.1	16,020
	*Apphia leucostriata*	PV796462.1	15,140
	*Apphia nigricarina*	NC_082034.1	16,090
	*Apphia nigricauda*	NC_082035.1	15,434
	*Apphia nigrinota*	PV762919.1	16,389
* Apphia *	*Apphia rudorsuma*	PV748917.1	15,508
	*Apphia rufipenna*	NC_077602.1	13,010
	*Apphia* sp. 1 SJ-2023a	NC_077603.1	13,115
	*Apphia* sp. 2 SJ-2025a	PV232320.1	15,076
* Carinata *	*Carinata dushanensis*	NC_077601.1	13,031
	*Carinata ganga*	NC_082036.1	15,025
	*Carinata recurvata*	NC_085760.1	15,277
	*Carinata rufipenna*	MN227165.1	15,261
* Chudania *	*Chudania hellerina*	MN227164.1	15,044
	*Chudania jinpinga*	PX412735.1	15,374
* Convexana *	*Convexana* sp. 1 JW-2022a	MW538525.1	15,096
	*Convexana vertebrana*	PX412732.1	14,857
* Cunedda *	*Cunedda* sp. 1 SJ-2023a	NC_077604.1	13,178
* Evacanthus *	*Evacanthus acuminatus*	MK948205.1	14,793
	*Evacanthus albicostatus*	PV796463.1	15,268
	*Evacanthus albimarginatus*	PX412728.1	14,827
	*Evacanthus albovittatus*	PX412730.1	16,617
	*Evacanthus biguttatus*	PV771628.1	17,452
	*Evacanthus bivittatus*	NC_077613.1	15,133
	*Evacanthus danmainus*	MN227166.1	15,343
	*Evacanthus densus*	PX412733.1	14,638
	*Evacanthus fatuus*	PV802422.1	14,863
	*Evacanthus fuscous*	PV802421.1	15,140
	*Evacanthus heimianus*	MN227167.1	15,680
	*Evacanthus* sp. 1 SJ-2025a	PV232321.1	15,382
* Onukia *	*Onukia* sp.	PX659856.1	15,572
	*Onukia abliclypeus*	KY264086.1	658
	*Onukia muirii*	KY264100.1	658
	*Onukia flavimacula*	KY264102.1	658
* Parapythamus *	*Parapythamus suiyangensis*	PV693707.1	15,252
* Striatanus *	*Striatanus curvatanus*	PV232324.1	15,589
	*Striatanus dentatus*	PV671704.1	14,974
	*Striatanus* sp. 1 SJ-2025a	PV232323.1	15,700
* Subulatus *	*Subulatus* sp.	OQ621798.1	14,858
	*Subulatus trimaculatus*	PX412729.1	15,451
* Taperus *	*Taperus fasciatus*	PX659855.1	15,262
* Transvenosus *	*Transvenosus sacculuses*	PV685812.1	16,004

### DNA extraction, sequencing, and genome assembly

Due to the small size of individual insects, multiple conspecific individuals collected from the same locality were pooled into a single mixed material for DNA extraction. Genomic DNA was isolated using the SanPrep DNA Gel Extraction Kit (Sangon Biotech, Shanghai, China). Genomic DNA extracted from each specimen underwent library preparation at Personalbio Inc. (Shanghai, China) and was sequenced on an Illumina platform. Raw reads were trimmed of adapters and quality-filtered to yield approximately 2.5 Gb of high-quality sequence data, which were assessed in DNASTAR SeqMan v.17.2 (Burland 2000) and subsequently *de novo* assembled in Geneious R9. 0 (Kearse et al. 2012).

### Mitochondrial genome annotation and feature analysis

The resulting mitochondrial genome assemblies were initially annotated using the MITOS v.2.1.9 web server with the invertebrate mitochondrial code (Bernt et al. 2013); annotations of protein-coding genes (PCGs) and ribosomal RNA genes were further validated by BLAST v.2.12.0 comparisons against NCBI (Altschul et al. 1997), and tRNA genes were confirmed through homology searches against published mitochondrial genomes. Intergenic regions and gene overlaps were manually inspected and delineated, while the secondary structures of 22 tRNA genes were predicted using tRNAscan-SE v.1.21 (Lowe and Eddy 1997) and ARWEN v.1.2. Annotated genomes were visualized with Proksee (https://proksee.ca/, accessed on 5 May 2025). For comparative genomic analyses, mitochondrial genome sequences from 38 Evacanthini species and two Nirvanini species (as outgroups) – a total of 40 taxa – were retrieved from GenBank and systematically evaluated for nucleotide composition and skew, PCG codon usage and relative synonymous codon usage (Gao et al. 2024), and overall genome architecture.

### Phylogenetic analysis

In this study, 40 mitochondrial genomes of Evacanthini and the outgroup Nirvanini were analyzed, including the newly sequenced *T. fasciatus* and *Onukia* sp. (GenBank accession numbers are provided in Table 1). After excising stop codons, the nucleotide sequences of 13 protein-coding genes (PCGs) and two rRNA genes were concatenated for phylogenetic reconstruction. Alignments were generated in MAFFT v.7.205 (Katoh and Standley 2013): each PCG was aligned codon-by-codon with the L-INS-i algorithm, whereas the two rRNAs were aligned with Q-INS-i to account for secondary structure constraints. Nucleotide saturation was assessed with DAMBE 5 and found to be non-significant; poorly aligned regions were removed using Gblocks v.0.91b with default settings (Talavera and Castresana 2007). The filtered alignments were concatenated in PhyloSuite v.1.2.3 (Zhang et al. 2019). Partition schemes and the best-fitting substitution models were selected with ModelFinder (Kalyaanamoorthy et al. 2017). Maximum-likelihood (**ML**) analysis was performed in IQ-TREE v.1.6.12 (Nguyen et al. 2014), with branch support evaluated by 5000 ultrafast bootstrap replicates. Bayesian inference (**BI**) was carried out in MrBayes v.3.2.6 (Ronquist et al. 2012), employing one cold and three heated chains in two independent MCMC runs of 2,000,000 generations each, sampling every 1000 generations and discarding the first 25% as burn-in. Convergence was accepted when the average standard deviation of split frequencies fell below 0.01, and all key parameters had effective sample sizes (**ESSs**) greater than 200. The final phylogenetic tree was visualized and taxonomically annotated in iTOL v.6.7.4 (Letunic and Bork 2021). The sequence alignment files generated and used for phylogenetic analyses in this study have been deposited in the Zenodo repository (https://doi.org/10.5281/zenodo.21336125) to facilitate data accessibility and reproducibility.

## Results

### General features of the mitochondrial genomes

**Figure 1.**
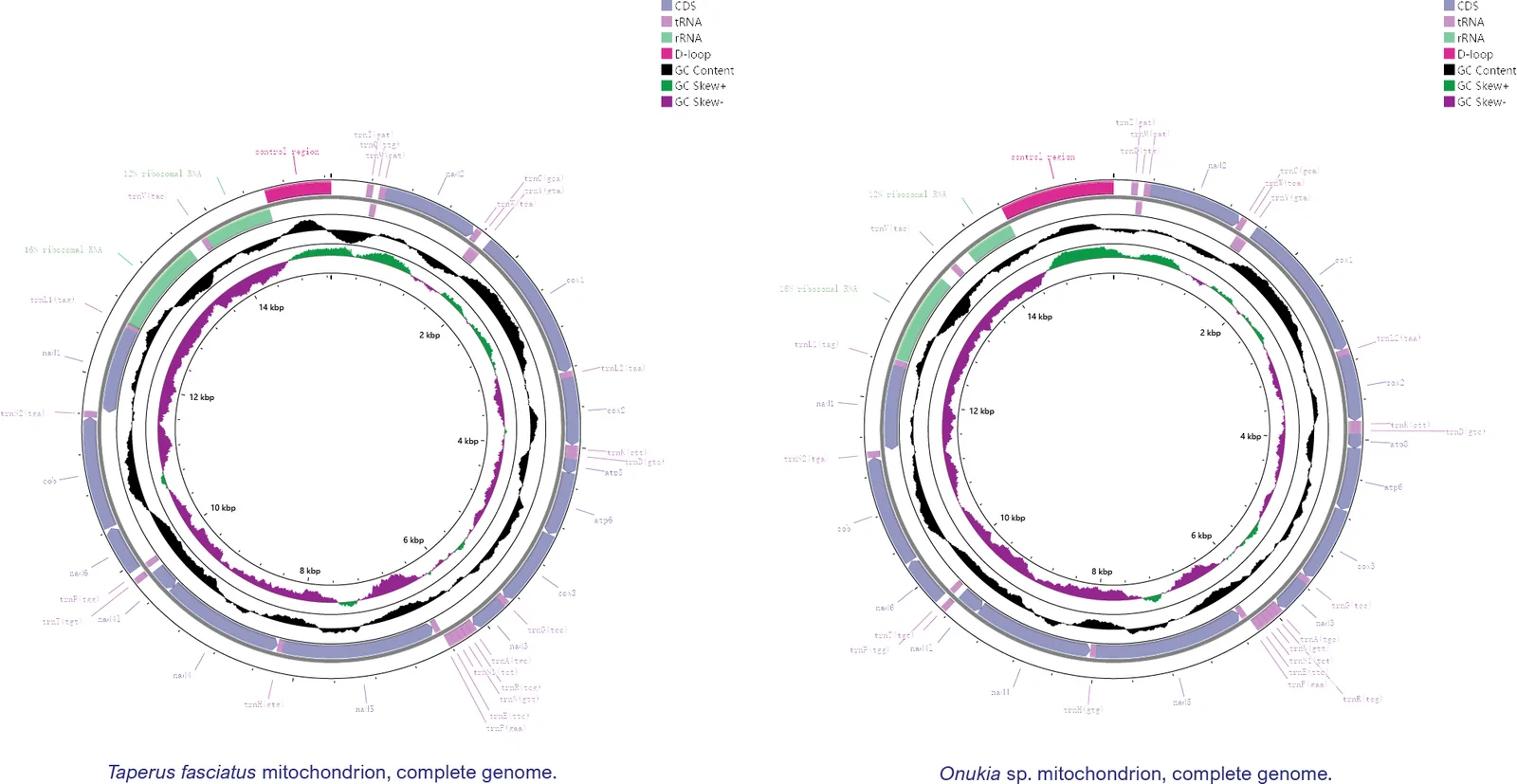
Mitogenomes of *T. fasciatus* and *Onukia* sp.

The complete mitochondrial genomes of *T. fasciatus* and *Onukia* sp. were successfully assembled and annotated. Both are closed circular molecules, with lengths of 15,262 bp and 15,572 bp, respectively. Each contains the standard 37 mitochondrial genes (13 PCGs, 22 tRNAs, 2 rRNAs) and a major non-coding control region. The gene arrangement order was completely consistent with that of most published Cicadellidae species, and no gene rearrangements were found. Most genes (23) were located on the major strand (J-strand), while the remaining 14 genes were on the minor strand (N-strand). (Fig. 1)

### Nucleotide composition and bias

Both genomes exhibited a strong AT bias. The overall A+T content was 79.4% for *T. fasciatus* and 78.2% for *Onukia* sp. The A+T content of PCGs (78.8% and 77.7%, respectively) was slightly lower than the whole-genome level, while tRNA and rRNA genes had higher A+T content, with the control region being the highest (>85%). At the whole-genome level, AT-skew values were positive (0.061 and 0.058), indicating a higher abundance of A - T; GC-skew values were negative (-0.084 and -0.081), indicating a higher abundance of C - G. This compositional bias was particularly pronounced at the third codon position (Table 2).

**Table 2.** Base composition of the mitochondrial genome of *T. fasciatus* and *Onukia* sp.

**Regions**	**Size (bp)**	**T(U)**	**C**	**A**	**G**	**AT(%)**	**GC(%)**	**AT skewness**	**GC skewness**
Full genome	15,262	37.3	11.1	42.1	9.4	79.4	20.5	0.061	-0.084
PCGs	10,926	46.2	10.1	32.6	11.2	78.8	21.3	-0.173	0.052
tRNAs	1414	40.3	8.2	40.2	11.2	80.5	19.4	-0.001	0.156
rRNAs	1777	46.5	6.8	34.9	11.7	81.4	18.5	-0.142	0.264
1^st^ codon position	3642	38.7	9.9	35.8	15.7	74.5	25.6	-0.038	0.226
2^nd^ codon position	3642	49.4	16.3	20.7	13.6	70.1	29.9	-0.409	-0.091
3^rd^ codon position	3642	50.4	4.1	41.1	4.3	91.5	8.4	-0.102	0.029
Full genome	15,572	36.8	11.7	41.4	10	78.2	21.7	0.058	-0.081
PCGs	10,929	45.6	10.6	32.1	11.7	77.7	22.3	-0.173	0.047
tRNAs	1431	40.5	8.2	39.9	11.5	80.4	19.7	-0.007	0.167
rRNAs	1731	46.2	7.2	34.3	12.2	80.5	19.4	-0.147	0.262
1^st^ codon position	3643	37.6	10.5	35.7	16.1	73.3	26.6	-0.027	0.212
2^nd^ codon position	3643	49.4	16.4	20.4	13.8	69.8	30.2	-0.415	-0.086
3^rd^ codon position	3643	49.6	5	40.3	5.1	89.9	10.1	-0.104	0.008

### Protein-coding genes and codon usage

As shown in Table 3, the total length of the 13 PCGs was 10,926 bp in *T. fasciatus* and 10,929 bp in *Onukia* sp. Except for *nad4L* in *T. fasciatus*, *atp8* in *Onukia* sp., and the *nad5* gene in both species, which uses TTG as the start codon, all other PCGs began with typical ATN codons (mainly ATG and ATT). Regarding stop codons, most genes used TAA or TAG, but the *cox1*, *cox2*, and *cox3* genes in both species terminated with only a single T nucleotide, presumably requiring post-transcriptional polyadenylation to form a complete stop signal.

**Table 3.** Mitogenomic organization of *T. fasciatus* and *Onukia* sp.

**Gene**	**Position**		**Size**	**Intergenic nucleotides**	**Codon**		**Strand**
	**From**	**To**			**Start**	**Stop**	
*Taperus fasciatus*/*Onukia* sp.							
trnI	359/183	423/249	65/67	358/182	–	–	H/H
trnQ	421/247	486/314	66/68	-3/-3	–	–	L/L
trnM	488/314	555/381	68/68	1/-1	–	–	H/H
nad2	556/382	1524/1350	969/969	–	ATA/ATA	TAA/TAA	H/H
trnW	1523/1349	1588/1411	66/63	-2/-2	–	–	H/H
trnC	1581/1404	1639/1463	59/60	-8/-8	–	–	L/L
trnY	1641/1465	1703/1531	63/67	1/1	–	–	L/L
cox1	1702/1532	3235/3065	1534/1534	-2/0	ATG/ATG	T/T	H/H
trnL2	3236/3066	3299/3129	64/64	–	–	–	H/H
cox2	3300/3130	3978/3808	679/679	–	ATT/ATT	T/T	H/H
trnK	3979/3809	4048/3878	70/70	–	–	–	H/H
trnD	4050/3878	4109/3940	60/63	1/-1	–	–	H/H
atp8	4112/3941	4264/4093	153/153	2/0	ATG/TTG	TAA/TAA	H/H
atp6	4258/4087	4905/4734	648/648	-7/-7	ATG/ATG	TAA/TAA	H/H
cox3	4908/4735	5685/5512	778/778	2/0	ATG/ATG	T/T	H/H
trnG	5685/5513	5750/5575	66/63	-1/0	–	–	H/H
nad3	5750/5576	6103/5929	354/354	-1/0	ATA/ATA	TAA/TAG	H/H
trnA	6104/5928	6164/5988	61/61	0/-2	–	–	H/H
trnR	6164/5989	6228/6051	65/63	-1/0	–	–	H/H
trnN	6227/6051	6290/6117	64/67	-2/-1	–	–	H/H
trnS1	6290/6117	6356/6182	67/66	-1/-1	–	–	H/H
trnE	6357/6183	6420/6247	64/65	–	–	–	H/H
trnF	6422/6249	6484/6312	63/64	1/1	–	–	L/L
nad5	6484/6312	8156/7985	1673/1674	-1/-1	TTG/TTG	TA/TAA	L/L
trnH	8157/7986	8219/8046	63/61	–	–	–	L/L
nad4	8222/8047	9541/9366	1320/1320	2/0	ATG/ATG	TAA/TAA	L/L
nad4L	9535/9360	9804/9629	270/270	-7/-7	TTG/ATG	TAA/TAA	L/L
trnT	9807/9632	9869/9697	63/66	2/2	–	–	H/H
trnP	9870/9698	9933/9762	64/65	–	–	–	L/L
nad6	9936/9765	10,418/10,247	483/483	2/2	ATT/ATT	TAA/TAA	H/H
cytb	10,432/10,253	11,568/11,389	1137/1137	13/5	ATG/ATG	TAG/TAA	H/H
trnS2	11,567/11,389	11,629/11,452	63/64	-2/-1	–	–	H/H
nad1	11,629/11,452	12,561/12,384	933/933	-1/-1	ATT/ATT	TAA/TAA	L/L
trnL1	12,562/12,385	12,627/12,448	66/64	–	–	–	L/L
rrnL	12,605/12,457	13,643/13,461	1039/1005	-23/8	–	–	L/L
trnV	13,799/13,613	13,862/13,684	64/72	155/151	–	–	L/L
rrnS	13,862/13,684	14,599/14,409	738/726	-1/-1	–	–	L/L

**Figure 2.**
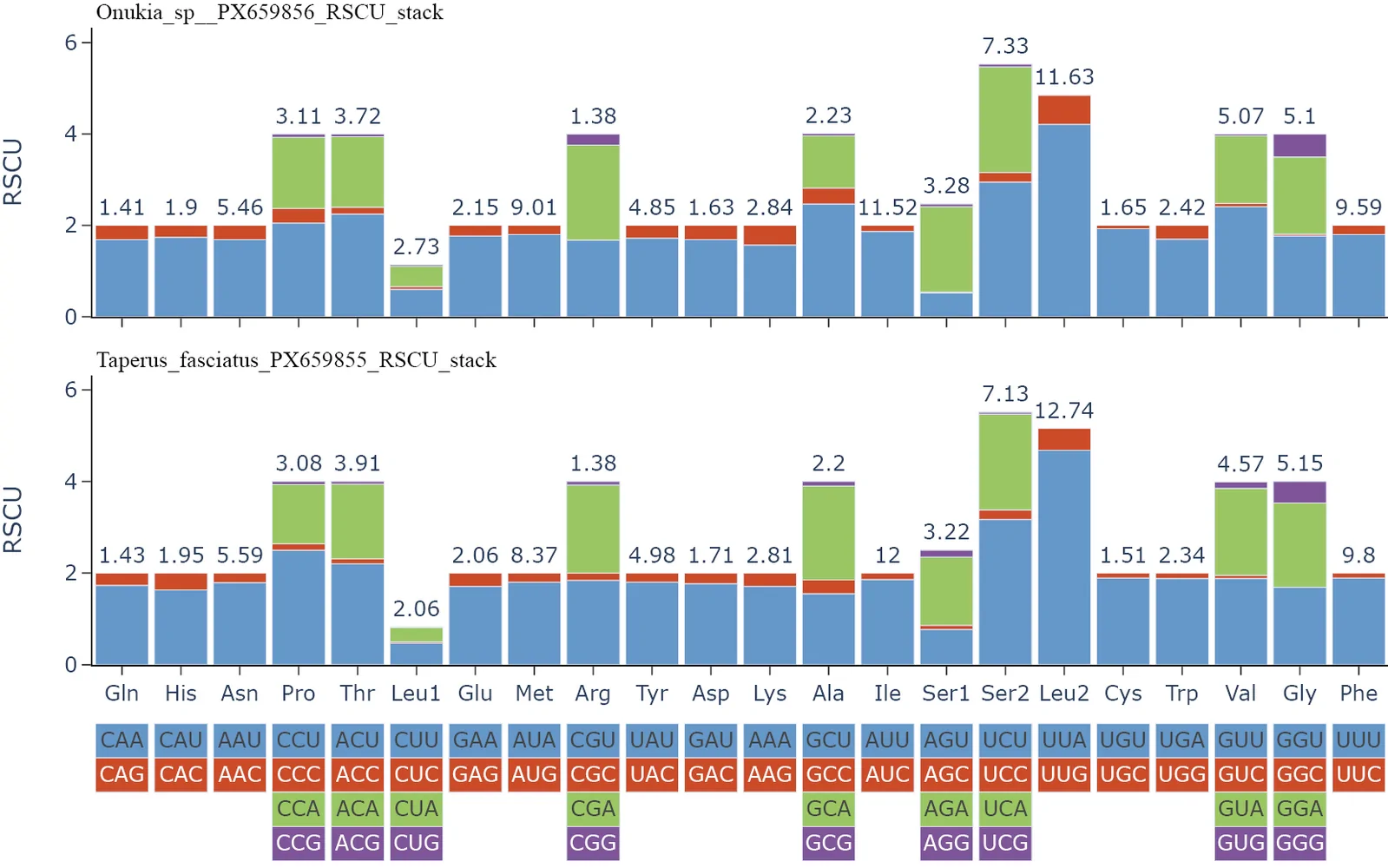
Relative synonymous codon usage (RSCU) of the mitogenome of *T. fasciatus* and *Onukia* sp.

**Figure 3.**
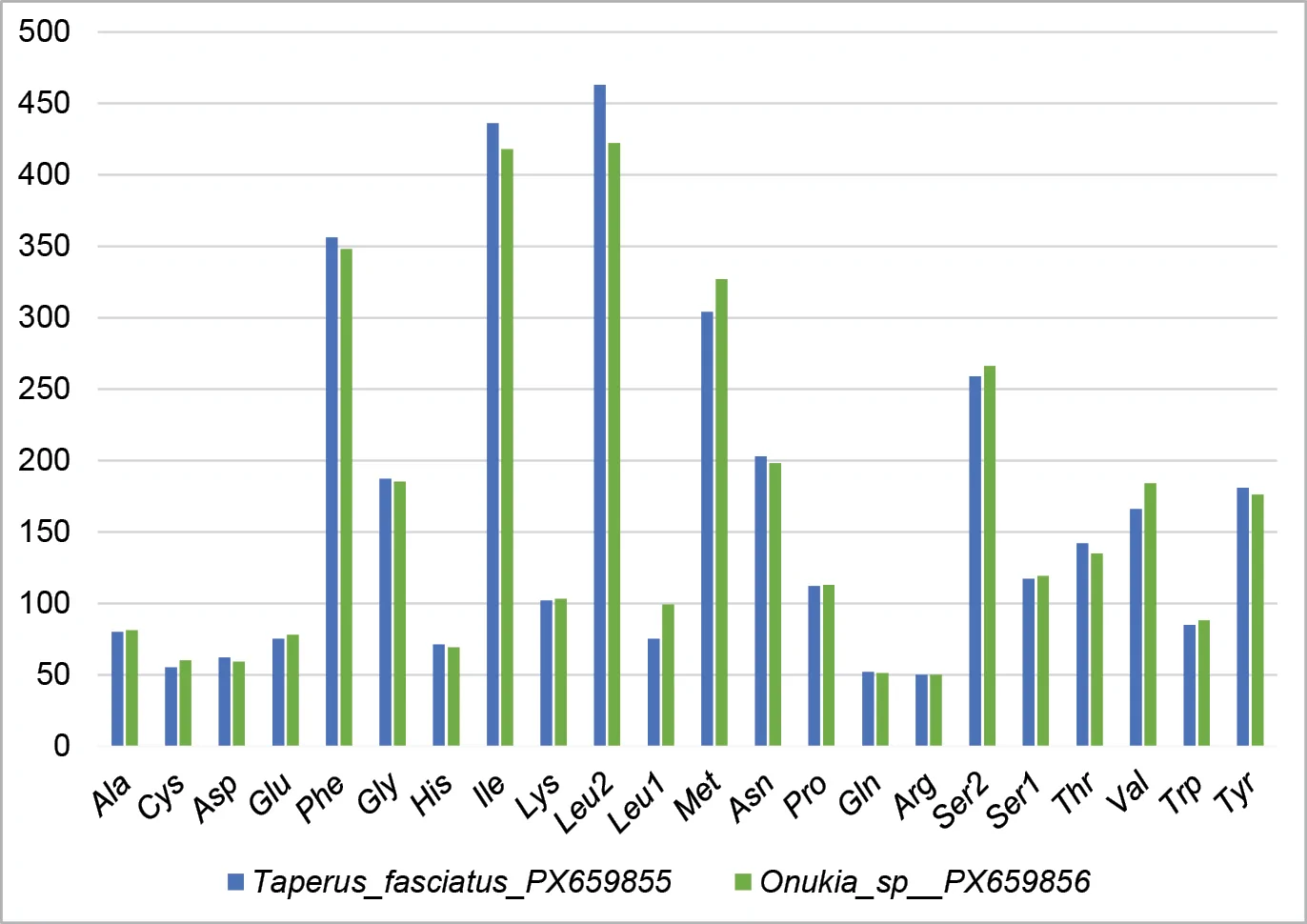
Amino acid composition analysis of protein-coding genes of *T. fasciatus* and *Onukia* sp.

RSCU (Fig. 2) analysis showed that the codon usage patterns of the two species were highly similar and exhibited a strong A/T bias. Codons ending with A or U (e.g., UUU/Phe, UUA/Leu, AUU/Ile) had RSCU values much greater than 1, indicating frequent usage, while those ending with C or G (e.g., CCC/Pro, GCC/Ala) had RSCU values much less than 1, indicating significant avoidance. Corresponding amino acid composition analysis (Fig. 3) revealed that Phenylalanine (Phe), Leucine (Leu), and Isoleucine (Ile) were the most abundant amino acids in the mitochondrial proteins.

### RNA genes

**Figure 4.**
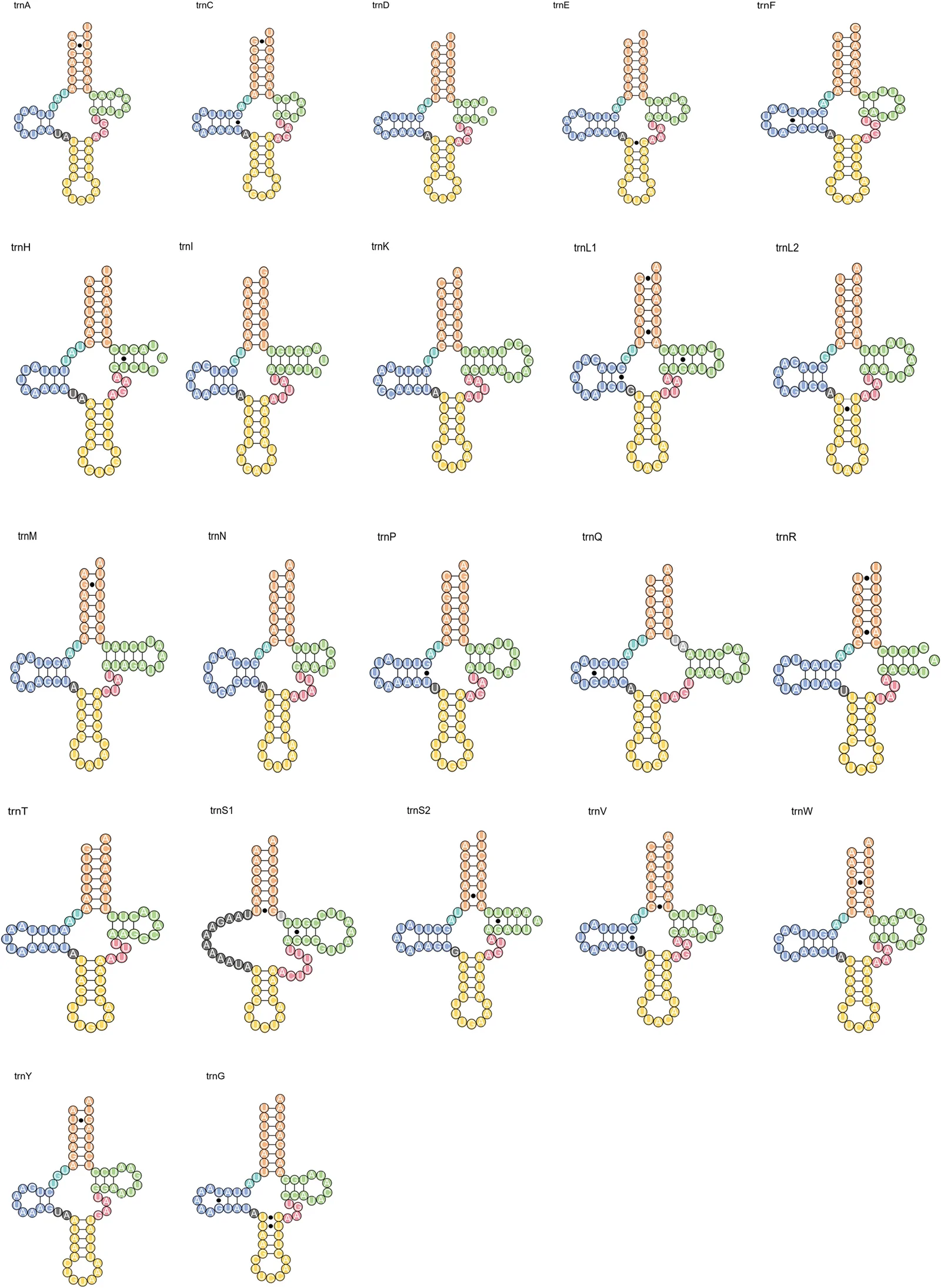
Secondary structure models for the 22 mitochondrial tRNAs encoded by the *T. fasciatus* mitogenome.

**Figure 5.**
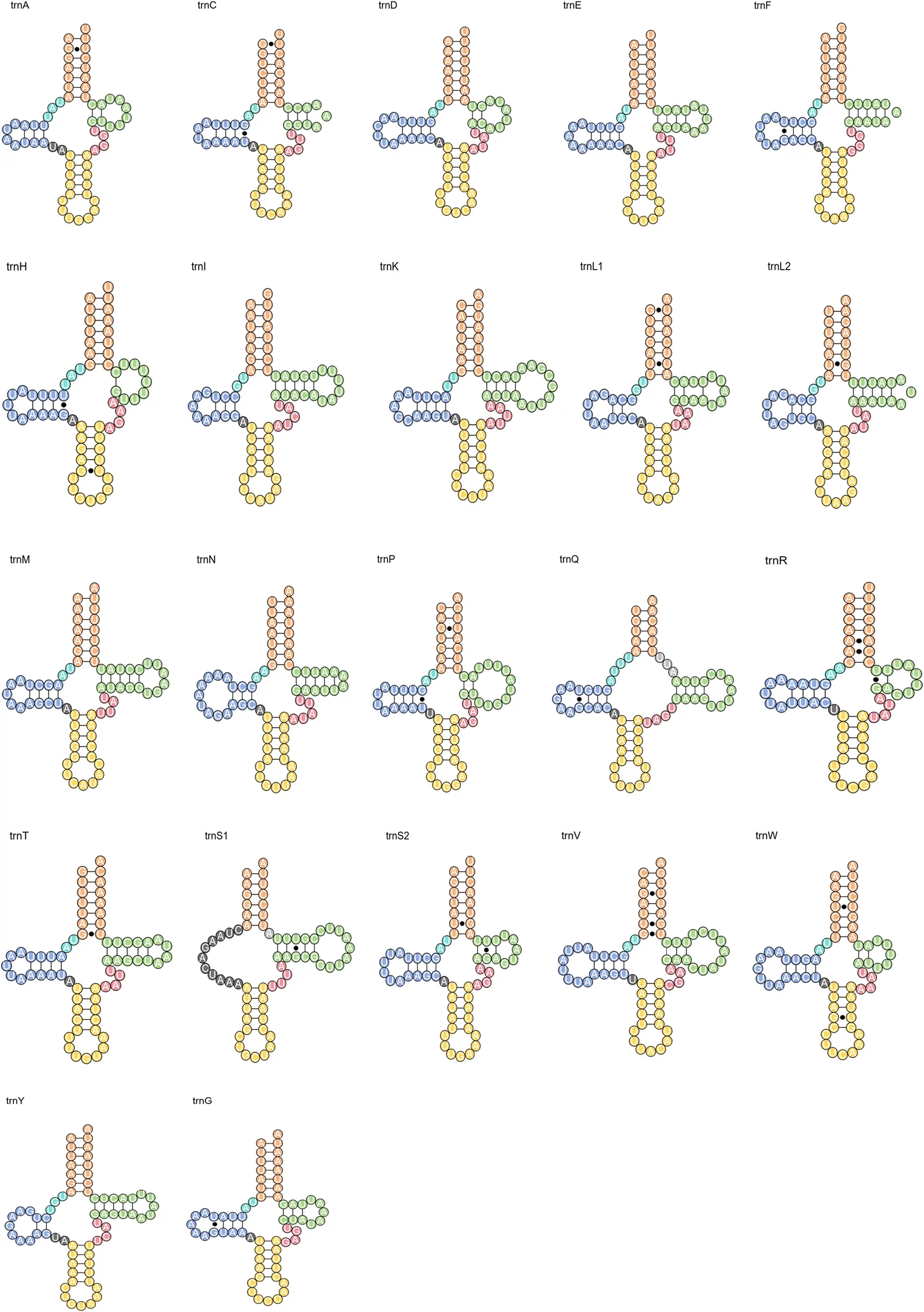
Secondary structure models for the 22 mitochondrial tRNAs encoded by the *Onukia* sp. mitogenome.

A total of 22 tRNA genes were predicted in both genomes, corresponding to the 20 standard amino acids (with two isoacceptor tRNAs each for Leucine and Serine). Except for trnS1(AGN), which formed a simplified secondary structure due to the lack of a complete DHU arm (Garey and Wolstenholme 1989), the remaining 21 tRNAs could be folded into the typical clover-leaf structure (Figs 4, 5). The lengths of the large (rrnL) and small (rrnS) ribosomal RNA genes were 1039 bp and 738 bp, respectively, for *T. fasciatus*, and 1005 bp and 546 bp, respectively, for *Onukia* sp. Their A+T content was the highest among the coding regions.

### Gene intergenic spacers and overlaps

Multiple gene intergenic spacers and overlapping regions were present in both genomes. *T. fasciatus* had 12 spacers totaling 540 bp; *Onukia* sp. had 8 spacers totaling 352 bp. Concurrently, *T. fasciatus* had 16 gene overlaps totaling 63 bp; *Onukia* sp. had 14 overlaps totaling 37 bp. Among these, the 7 bp overlap between ATP8 and ATP6 is a highly conserved feature in insect mitochondrial genomes. Additionally, a conserved 3 bp overlap was found between trnI and trnQ.

### Control region

The control region, also called the A+T-rich region, was the longest non-coding region in these mitogenomes. This region is located between rrnS and trnI, and its size varies, ranging from 518 bp (*U. puerana* Yang & Meng, 2010) to 1610 bp (*M. ponta* Yang & Li, 1999). The control region was 663 bp long in *T. fasciatus* and 1163 bp long in *Onukia* sp. They all have a mild positive or negative AT-skew and GC-skew.

### Phylogenetic analysis

**Figure 6.**
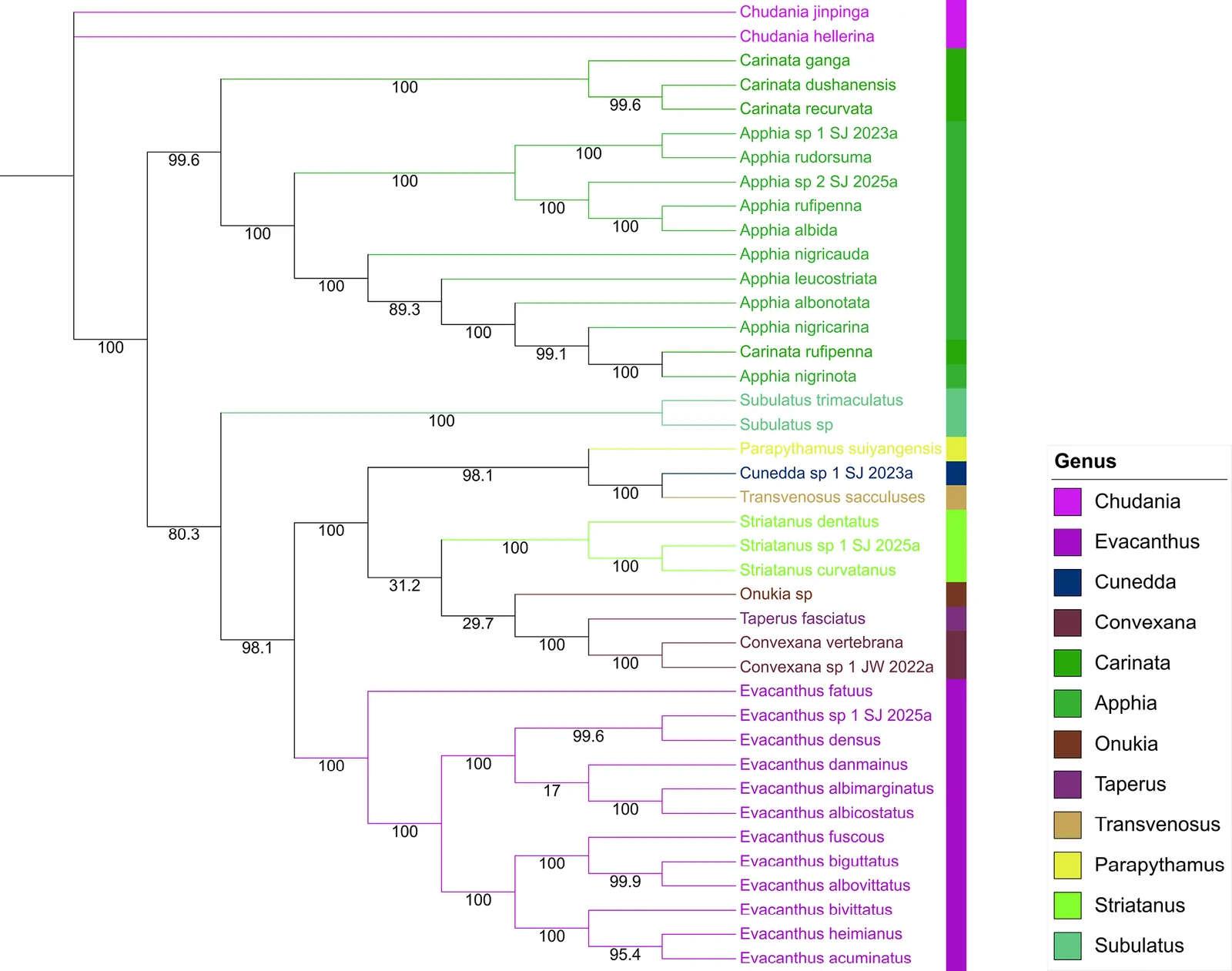
Phylogenetic relationships of Evacanthini species inferred from maximum likelihood (ML) analysis based on complete mitogenome sequences. Numbers on the branches indicate bootstrap support values.

**Figure 7.**
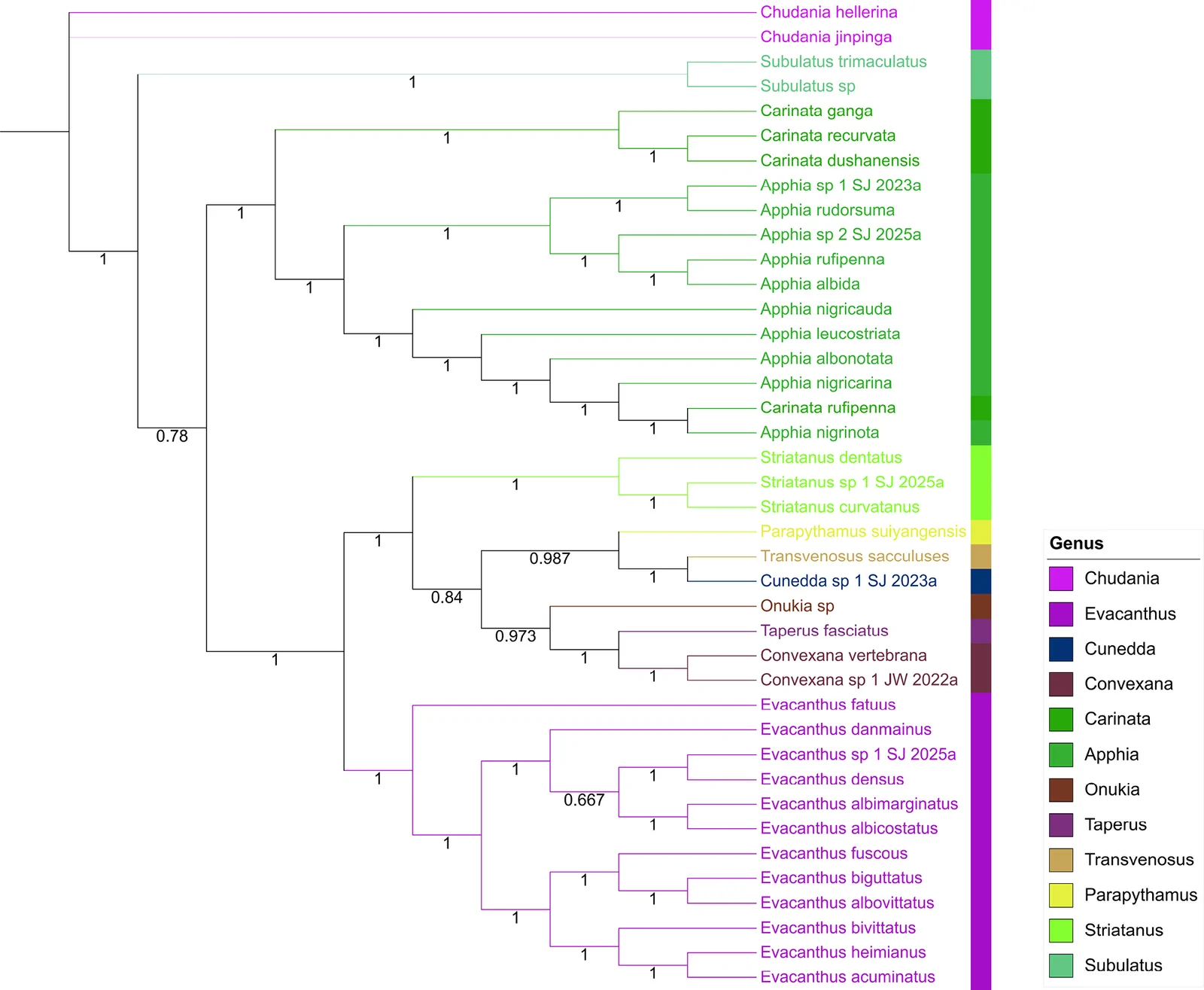
Phylogenetic relationships of Evacanthini species inferred from Bayesian inference (BI) analysis based on complete mitogenome sequences. Numbers on the branches indicate Bayesian posterior probabilities.

Phylogenetic analyses based on a concatenated dataset of 13 protein-coding genes (PCGs) and 2 rRNA genes using maximum likelihood (ML) (Fig. 6) and Bayesian inference (BI) (Fig. 7) produced highly congruent topologies with generally strong node support, indicating robust phylogenetic inference. Both analyses strongly support the monophyly of Evacanthini. The outgroup (Nirvanini) is consistently placed at the base of the trees, confirming its basal position relative to the tribe. Within Evacanthini, all major genera form independent, well-supported monophyletic clades, indicating that the tribe constitutes a coherent and evolutionarily distinct lineage.

Within the tribe, *Onukia* sp. and *T. fasciatus* occupy separate branches and do not form a sister-group relationship in either tree. The ML tree shows low support for the corresponding node (BS = 29.7), while the BI tree gives a slightly higher posterior probability (PP = 0.973), still lower than that of the core clades (PP = 1). This suggests that their phylogenetic positions within the tribe are uncertain. Both taxa are positioned closer to the genus *Convexana* Li, 1994 rather than constituting a sister-group relationship with one another.

By contrast, multi-species genera such as *Apphia* Distant, 1918 and *Evacanthus* Le Peletier & Audinet-Serville, 1828 form strongly supported monophyletic clades in both analyses, with stable internal topologies that reflect well-resolved intra-generic relationships. Notably, *Carinata rufipenna* Li & Wang, 1992 clusters with several *Apphia* species in both ML and BI trees, whereas the remaining *Carinata* Li & Wang, 1992 species (*C. recurvata* Wang & Li, 1998, *C. dushanensis* Li, Li & Xing, 2020, *C. ganga* Li & Wang, 1992) form a well-supported monophyletic group. This atypical pattern may reflect multiple factors, including conserved or convergent mitochondrial sequences, ancestral mitochondrial polymorphism, early rapid radiation events, or minor sampling and taxonomic uncertainties. The placement of *C. rufipenna* among *Apphia* species highlights the complex evolutionary history and interlaced lineages within Evacanthini.

Overall, these results not only corroborate the monophyly of Evacanthini but also reveal potential phylogenetic complexities within certain genera, providing a valuable framework for future taxonomic revisions and phylogenetic investigations.

**Figure 8.**
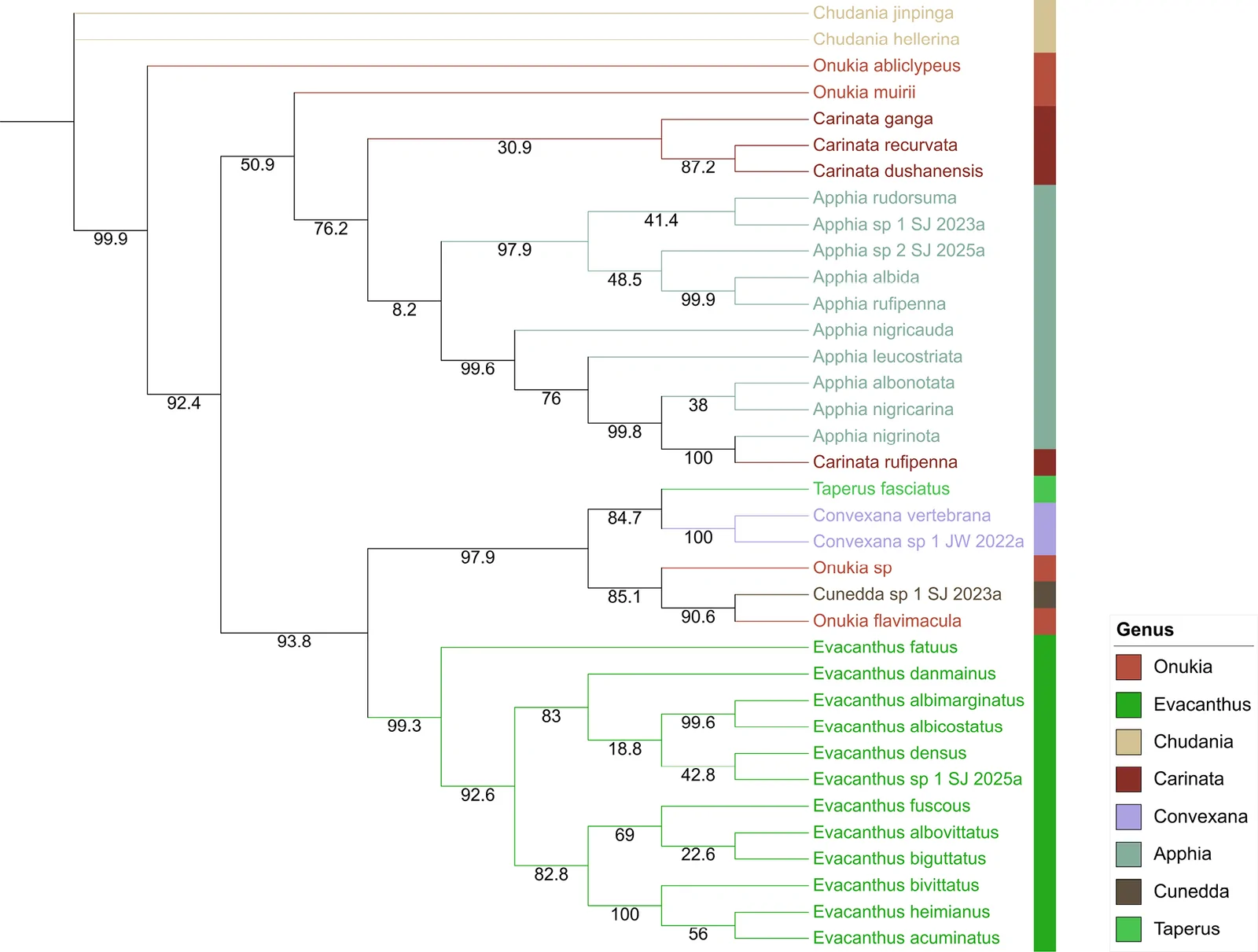
Phylogenetic relationships of Evacanthini species inferred from maximum likelihood (ML) analysis based on partial mitochondrial *cox1* sequences. Numbers on the branches indicate bootstrap support values.

To further test the hypothesis that *Onukia* is not monophyletic, we reconstructed a phylogenetic tree using maximum likelihood (ML) based on partial mitochondrial *cox1* sequences from 36 species (Fig. 8). The resulting tree shows that species of *Onukia* do not form a single monophyletic clade, but are distributed across multiple branches in proximity to different genera. These findings further support the hypothesis proposed by Wang et al. that the genus is non-monophyletic.

Nonetheless, there are limitations to this study. Due to the scarcity of complete mitochondrial genomes for *Onukia*, only partial *cox1* sequences could be used, which may not fully capture deeper phylogenetic signals or resolve complex relationships among closely related species. Therefore, although the results support the non-monophyly of *Onukia*, additional molecular data and broader taxon sampling will be necessary to fully resolve genus-level relationships and patterns of intra-generic diversification.

Importantly, this study provides the first complete mitochondrial genome for the genus, laying a foundation for more precise assessments of the phylogenetic relationships within *Onukia* in future research.

## Discussion

This study presents the first complete mitochondrial genomes of *T. fasciatus* and *Onukia* sp., providing critical molecular resources for the leafhopper tribe Evacanthini. Comparative analyses indicate that these mitogenomes exhibit typical Cicadellidae features (Perna and Kocher 1995; Boore 1999), including conserved gene order, high A+T content, overlapping regions between *atp8* and *atp6*, and the absence of a DHU arm in *trnS1*(AGN), consistent with previous observations in other Hemiptera (Boore 1999; Wu et al. 2015; Luo et al. 2019). Inter-species variation in the control region length and the use of alternative start codons (e.g., TTG) may provide phylogenetic signals at the genus and species level (Cameron 2014; Demari-Silva et al. 2015; Du et al. 2021), highlighting the utility of mitochondrial genomes for resolving evolutionary relationships among closely related taxa (Cameron 2014; Du et al. 2021).

Phylogenetic analyses based on a concatenated dataset of 13 protein-coding genes and two rRNA genes, using both maximum likelihood (ML) and Bayesian inference (BI) approaches, strongly supported the monophyly of Evacanthini (Dietrich 2004; Jiang et al. 2024). Major genera within the tribe, such as *Apphia* and *Evacanthus*, formed well-supported monophyletic clades with stable internal topologies, indicating relatively consistent evolutionary relationships. The newly sequenced mitochondrial genomes of *T. fasciatus* and *Onukia* sp. further expanded the molecular resources available for Evacanthini and provided additional insights into the phylogenetic relationships of these genera. In our mitogenomic tree, *T. fasciatus* was closely associated with *Convexana*, supporting the close relationship between *Taperus* Li & Wang, 1994 and *Convexana*. In contrast, *Onukia* sp. exhibited a relatively unstable phylogenetic position and did not form a strongly supported sister-group relationship with *T. fasciatus*. Instead, *Onukia* sp. was recovered outside the clade containing *Taperus* and *Convexana*. This topology received low support in ML analysis (BS = 29.7) but relatively high Bayesian posterior probability (PP = 0.973), suggesting that the precise phylogenetic placement of *Onukia* sp. requires further investigation with additional data. However, *Carinata rufipenna* showed an unexpected placement among several *Apphia* species, which may reflect differences in phylogenetic signals among mitochondrial datasets, ancestral polymorphism, or rapid diversification events (Cameron 2014; Du et al. 2021), highlighting the complex evolutionary history within Evacanthini.

Comparison with the phylogenetic hypothesis of Wang et al. (2017) revealed both similarities and differences in the evolutionary relationships within Evacanthini. Both studies strongly supported the monophyly of Evacanthini and recovered *Apphia* and *Evacanthus* as relatively stable monophyletic lineages. In addition, the close relationship between *Taperus* and *Convexana* was generally consistent between the two studies. However, differences in the placement of some taxa were observed; these discrepancies may be associated with differences in molecular datasets, taxon sampling, and analytical approaches. Wang et al. (2017) inferred phylogenetic relationships using a combined dataset comprising mitochondrial *cox1*, nuclear genes (28S, H3, and wingless), and morphological characters, whereas the present study utilized complete mitochondrial genome data comprising 13 protein-coding genes and two rRNA genes. The different types of phylogenetic information provided by these datasets may contribute to variations in the resolution of certain relationships.

In addition, a *cox1*-based analysis was conducted to further evaluate the phylogenetic status of *Onukia*. Consistent with Wang et al. (2017), our results did not recover *Onukia* as a monophyletic genus. The inclusion of the newly sequenced *Onukia* species did not alter this pattern, with different *Onukia* species distributed among multiple lineages rather than forming a single clade. Although the detailed placement of individual species differed between the two analyses, these differences may result from variations in molecular markers and taxon sampling. Therefore, our results provide additional support for the hypothesis that the current classification of *Onukia* may not accurately reflect its evolutionary history.

Despite these insights, limitations remain. The limited availability of complete mitochondrial genomes for *Onukia* restricts a comprehensive evaluation of its genus-level phylogenetic relationships. In addition, mitochondrial datasets alone may not fully resolve deeper evolutionary relationships or complex diversification patterns. Future studies should integrate additional mitochondrial and nuclear genomic data, together with broader taxon sampling (Li et al. 2025), to improve phylogenetic resolution, account for incomplete lineage sorting, and further clarify evolutionary relationships within Evacanthini (Wang et al. 2017; Jiang et al. 2024).

Collectively, this study supports the monophyly of Evacanthini based on complete mitochondrial genome data and provides new insights into the evolutionary relationships among its constituent genera. The newly sequenced mitochondrial genomes represent valuable molecular resources for future taxonomic revisions, phylogenetic investigations, and integrative morphological–molecular analyses, contributing to a more comprehensive understanding of the evolutionary diversity and systematics of this agriculturally and ecologically important leafhopper tribe (Dietrich 2004; Wang et al. 2017).

## Additional information

### Artificial Intelligence (AI) use

The authors accept full responsibility for the content of the manuscript, including the disclosure of any use of AI.

No AI tools were used in the preparation of this manuscript.

### Author ORCIDs

Y. Li https://orcid.org/0009-0007-5750-5922

L. Jiang https://orcid.org/0000-0003-3217-9191

S. Jiang https://orcid.org/0000-0001-5084-1295

Y. Guo https://orcid.org/0009-0007-6981-3682

R. Li https://orcid.org/0000-0001-8567-3527

J. Xing https://orcid.org/0000-0002-3738-7996

B. Zhang https://orcid.org/0000-0001-8702-9022

Y. Li https://orcid.org/0000-0002-0470-203X

### Data availability

The data supporting the findings of this study are openly available from the National Center for Biotechnology Information at https://www.ncbi.nlm.nih.gov, accession numbers: PX659855.1, PX659856.1.

## References

[B1] Aguin-Pombo D, Aguiar AMF, Kuznetsova VG (2007) Bionomics and taxonomy of leafhopper *Sophonia orientalis* (Homoptera: Cicadellidae), a Pacific pest species in the Macaronesian Archipelagos. Annals of the Entomological Society of America 100: 19–26. 10.1603/0013-8746(2007)100[19:batols]2.0.co;2

[B2] Altschul SF, Madden TL, Schäffer AA, Zhang J, Zhang Z, Miller W, Lipman DJ (1997) Gapped BLAST and PSI-BLAST: a new generation of protein database search programs. Nucleic Acids Research 25: 3389–3402. 10.1093/nar/25.17.3389 PMC146917 9254694

[B3] Ballard JW, Whitlock MC (2004) The incomplete natural history of mitochondria. Molecular Ecology 13: 729–744. 10.1046/j.1365-294x.2003.02063.x 15012752

[B4] Bernt M, Donath A, Jühling F, Externbrink F, Florentz C, Fritzsch G, Pütz J, Middendorf M, Stadler PF (2013) MITOS: Improved *de novo* metazoan mitochondrial genome annotation. Molecular Phylogenetics and Evolution 69: 313–319. 10.1016/j.ympev.2012.08.023 22982435

[B5] Boore JL (1999) Animal mitochondrial genomes. Nucleic Acids Research 27: 1767–1780. 10.1093/nar/27.8.1767 PMC148383 10101183

[B6] Burland TG (2000) DNASTAR’s Lasergene Sequence Analysis Software. In: Misener S, Krawetz SA (Eds) Bioinformatics Methods and Protocols. Humana Press, 71–91. 10.1385/1-59259-192-2:71 10547832

[B7] Cameron SL (2014) Insect mitochondrial genomics: implications for evolution and phylogeny. Annual Review of Entomology 59: 95–117. 10.1146/annurev-ento-011613-162007 24160435

[B8] Chen X, Yang L, Li Z (2012) Bamboo-Feeding Leafhoppers in China. Forestry Publishing House, Beijing, 218 pp.

[B9] Demari-Silva B, Foster PG, de Oliveira TMP, Bergo ES, Sanabani SS, Pessôa R, Sallum MAM (2015) Mitochondrial genomes and comparative analyses of *Culex camposi*, *Culex coronator*, *Culex usquatus* and *Culex usquatissimus* (Diptera: Culicidae), members of the coronator group. BMC Genomics 16: 831. 10.1186/s12864-015-1951-0 PMC4618934 26489754

[B10] Dietrich CH (2004) Phylogeny of the leafhopper subfamily Evacanthinae with a review of Neotropical species and notes on related groups (Hemiptera: Membracoidea: Cicadellidae). Systematic Entomology 29: 455–487. 10.1111/j.0307-6970.2004.00250.x

[B11] Dietrich CH, Allen JM, Lemmon AR, Lemmon EM, Takiya DM, Evangelista O, Walden KKO, Grady PGS, Johnson KP (2017) Anchored hybrid enrichment-based phylogenomics of leafhoppers and treehoppers (Hemiptera: Cicadomorpha: Membracoidea). Insect Systematics and Diversity 1: 57–72. 10.1093/isd/ixx003

[B12] Du Y, Liang Z, Dietrich CH, Dai W (2021) Comparative analysis of mitochondrial genomes of Nirvanini and Evacanthini (Hemiptera: Cicadellidae) reveals an explicit evolutionary relationship. Genomics 113: 1378–1385. 10.1016/j.ygeno.2021.03.017 33716186

[B13] Gao W, Chen X, He J, Sha A, Luo Y, Xiao W, Xiong Z, Li Q (2024) Intraspecific and interspecific variations in the synonymous codon usage in mitochondrial genomes of 8 pleurotus strains. BMC Genomics 25: 456. 10.1186/s12864-024-10374-3 PMC11084086 38730418

[B14] Garey JR, Wolstenholme DR (1989) Platyhelminth mitochondrial DNA: Evidence for early evolutionary origin of a tRNAserAGN that contains a dihydrouridine arm replacement loop, and of serine-specifying AGA and AGG codons. Journal of Molecular Evolution 28: 374–387. 10.1007/bf02603072 2545889

[B15] Hu Y, Dietrich CH, Skinner RK, Zhang Y (2022) Phylogeny of Membracoidea (Hemiptera: Auchenorrhyncha) based on transcriptome data. Systematic Entomology 48: 97–110. 10.1111/syen.12563

[B16] Jiang S, Jiang L, Li R, Gao H, Zhang A, Yan Y, Zou X, Wu J, Xu S, Yi X, Li Y (2022) The complete mitochondrial genome of *Coccotorus beijingensis* Lin et Li, 1990 (Coleoptera, Curculionidae, Curculioninae, Anthonomini) and its phylogenetic implications. Biodiversity Data Journal 10: e95935. 10.3897/BDJ.10.e95935 PMC9836419 36761507

[B17] Jiang S, Li R, Jiang L, Wang W, Liu Y, Yang Y, Xing J, Li Z, Li Y (2024) The complete mitochondrial genomes of three Evacanthini species, *Evacanthus bivittatus*, *Carinata ganga*, and *Carinata recurvata*, and phylogenomic analysis of the Evacanthini. Frontiers in Ecology and Evolution 12: 1410546. 10.3389/fevo.2024.1410546

[B18] Kalyaanamoorthy S, Minh BQ, Wong TKF, von Haeseler A, Jermiin LS (2017) ModelFinder: fast model selection for accurate phylogenetic estimates. Nature Methods 14: 587–589. 10.1038/nmeth.4285 PMC5453245 28481363

[B19] Katoh K, Standley DM (2013) MAFFT Multiple Sequence Alignment Software Version 7: Improvements in performance and usability. Molecular Biology and Evolution 30: 772–780. 10.1093/molbev/mst010 PMC3603318 23329690

[B20] Kearse M, Moir R, Wilson A, Stones-Havas S, Cheung M, Sturrock S, Buxton S, Cooper A, Markowitz S, Duran C, Thierer T, Ashton B, Meintjes P, Drummond A (2012) Geneious Basic: An integrated and extendable desktop software platform for the organization and analysis of sequence data. Bioinformatics 28: 1647–1649. 10.1093/bioinformatics/bts199 PMC3371832 22543367

[B21] Letunic I, Bork P (2021) Interactive Tree Of Life (iTOL) v5: an online tool for phylogenetic tree display and annotation. Nucleic Acids Research 49: W293–W296. 10.1093/nar/gkab301 PMC8265157 33885785

[B22] Li Y, Guo Y, Li R, Liu Y, Xue C, Jiang L, Jiang S, Wang W, Yi X (2025) The complete mitochondrial genome of *Petalocephala arcuata* Cai et Kuoh, 1992 (Hemiptera: Cicadellidae: Ledrinae: Petalocephalini) and its phylogenetic implications. Genes 16: 567. 10.3390/genes16050567 PMC12110874 40428389

[B23] Li Z, Li Y, Xing J (2020) Fauna Sinica: Insecta Vol. 72: Hemiptera: Cicadellidae (IV): Evacanthunae. Science Press, Beijing, 1–547.

[B24] Lowe TM, Eddy SR (1997) tRNAscan-SE: A program for improved detection of transfer RNA genes in genomic sequence. Nucleic Acids Research 25: 955–964. 10.1093/nar/25.5.955 PMC146525 9023104

[B25] Luo X, Chen Y, Chen C, Pu D, Tang X, Zhang H, Lu D, Mao J (2019) Characterization of the complete mitochondrial genome of *Empoasca* sp. (Cicadellidae: Hemiptera). Mitochondrial DNA, Part B, 4: 1477–1478. 10.1080/23802359.2019.1579066

[B26] Nguyen L-T, Schmidt HA, von Haeseler A, Minh BQ (2014) IQ-TREE: A fast and effective stochastic algorithm for estimating maximum-likelihood phylogenies. Molecular Biology and Evolution 32: 268–274. 10.1093/molbev/msu300 PMC4271533 25371430

[B27] Perna NT, Kocher TD (1995) Patterns of nucleotide composition at fourfold degenerate sites of animal mitochondrial genomes. Journal of Molecular Evolution 41: 353–358. 10.1007/BF00186547 7563121

[B28] Ronquist F, Teslenko M, van der Mark P, Ayres DL, Darling A, Höhna S, Larget B, Liu L, Suchard MA, Huelsenbeck JP (2012) MrBayes 3.2: efficient Bayesian phylogenetic inference and model choice across a large model space. Systematic Biology 61: 539–542. 10.1093/sysbio/sys029 PMC3329765 22357727

[B29] Taanman J-W (1999) The mitochondrial genome: structure, transcription, translation and replication. Biochimica et Biophysica Acta (BBA) - Bioenergetics 1410: 103–123. 10.1016/s0005-2728(98)00161-3 10076021

[B30] Talavera G, Castresana J (2007) Improvement of phylogenies after removing divergent and ambiguously aligned blocks from protein sequence alignments. Systematic Biology 56: 564–577. 10.1080/10635150701472164 17654362

[B31] Wang W, Jiang L, Li R, Jiang S, Liu Y, Xing J, Li Y (2024) The complete mitochondrial genomes of five Nivanini species (Hemiptera: Cicadellidae: Evacanthinae) with phylogenetic analysis. Ecology and Evolution 14: e70413. 10.1002/ece3.70413 PMC11470156 39398627

[B32] Wang X, Wang J, Dai R (2021) Mitogenomics of five *Olidiana* leafhoppers (Hemiptera: Cicadellidae: Coelidiinae) and their phylogenetic implications. PeerJ 9: e11086. 10.7717/peerj.11086 PMC8086571 33986976

[B33] Wang Y, Dietrich CH, Zhang Y (2017) Phylogeny and historical biogeography of leafhopper subfamily Evacanthinae (Hemiptera: Cicadellidae) based on morphological and molecular data. Scientific Reports 7: 45387. 10.1038/srep45387 PMC5377372 28368039

[B34] Wilson AC, Cann RL, Carr SM, George M, Gyllensten UB, Helm-Bychowski KM, Higuchi RG, Palumbi SR, Prager EM, Sage RD, Stoneking M (1985) Mitochondrial DNA and two perspectives on evolutionary genetics. Biological Journal of the Linnean Society 26: 375–400. 10.1111/j.1095-8312.1985.tb02048.x

[B35] Wolstenholme DR (1992) Animal Mitochondrial DNA: Structure and Evolution. In: Wolstenholme DR, Jeon KW (Eds) International Review of Cytology. Elsevier, Volume 141, 173–216. 10.1016/s0074-7696(08)62066-5 1452431

[B36] Wu Y, Dai R, Zhan H, Qu L (2015) Complete mitochondrial genome of *Drabescoides nuchalis* (Hemiptera: Cicadellidae). Mitochondrial DNA, Part A, 27: 3626–3627. 10.3109/19401736.2015.1079827 26436567

[B37] Zhang D, Gao F, Jakovlić I, Zou H, Zhang J, Li WX, Wang GT (2019) PhyloSuite: An integrated and scalable desktop platform for streamlined molecular sequence data management and evolutionary phylogenetics studies. Molecular Ecology Resources 20(1): 348–355. 10.1111/1755-0998.13096 31599058

